# Supplementary figures and images for: Protection of H2S against Hypoxia/Reoxygenation Injury in Rat Hippocampal Neurons through Inhibiting Phosphorylation of ROCK2 at Thr436 and Ser575
Source: Pharmaceuticals (Basel). 2023 Jan 31;16(2):218. doi: 10.3390/ph16020218 (PMC9966024; doi:10.3390/ph16020218)

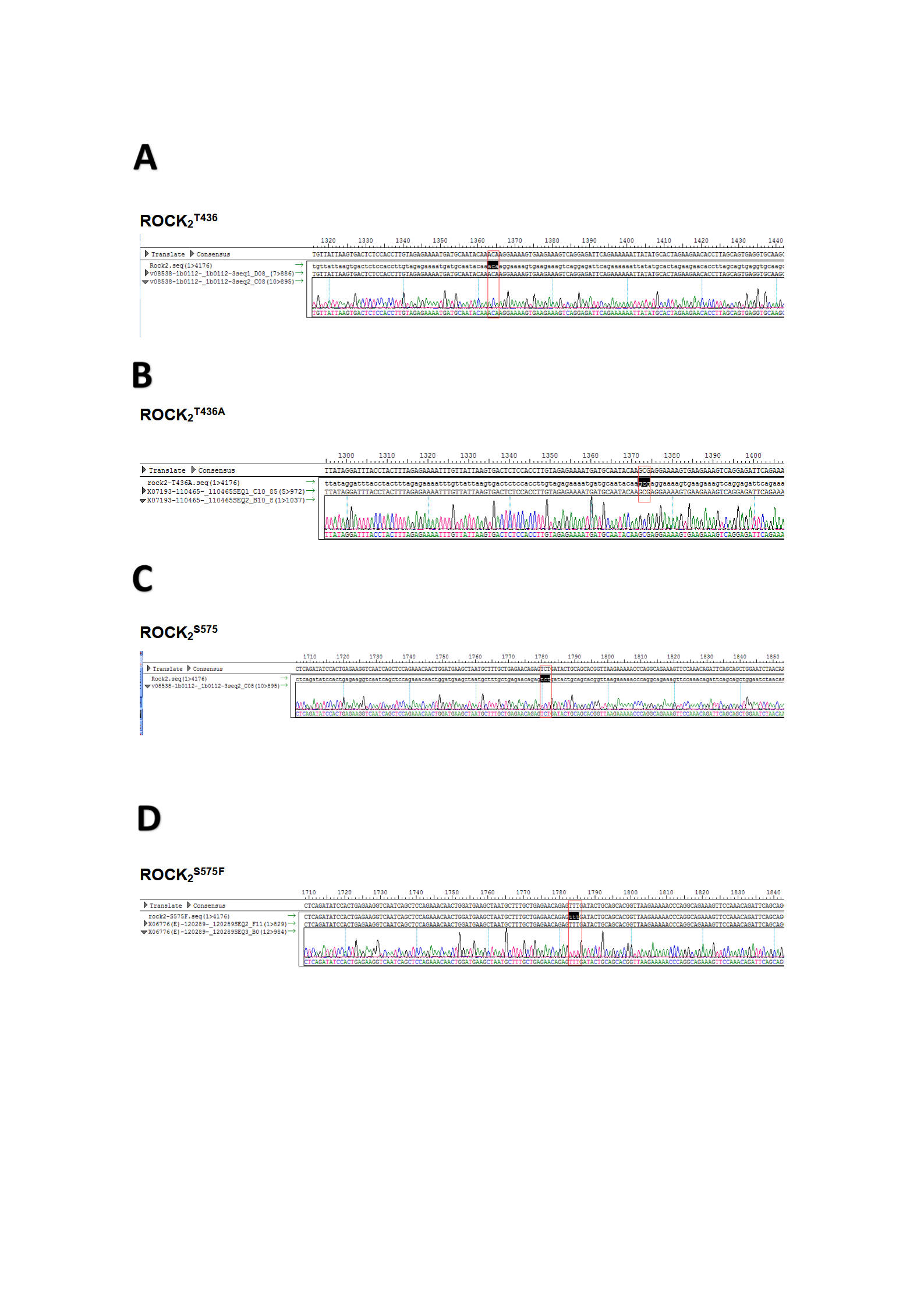

Supplement: Supplementary file 1 [file pharmaceuticals-16-00218-s001.zip › supplementary material.tif]
